# Powdery Scab of Potato: An Evaluation of Current Molecular Resources

**DOI:** 10.1111/1758-2229.70295

**Published:** 2026-02-08

**Authors:** Sadegh Balotf, Calum Wilson

**Affiliations:** ^1^ Centre for Crop Health University of Southern Queensland Toowoomba Queensland Australia; ^2^ Tasmanian Institute of Agriculture University of Tasmania Hobart Tasmania Australia

**Keywords:** genomic, metabolomic, potato, proteomic, *Spongospora subterranea* f. sp. *Subterranea*, transcriptomic

## Abstract

*Spongospora subterranea* f. sp. *Subterranea* (Sss), the causal agent of powdery scab in potatoes, is a globally important soil‐borne pathogen with an obligate biotrophic lifestyle and long‐lived resting spores that make it difficult to control. This paper compiles and evaluates all currently available molecular resources for Sss, including genomic, transcriptomic, proteomic, and metabolomic datasets, providing the first comprehensive overview of the pathogen's molecular research landscape. Although recent long‐read assemblies have advanced the Sss genome, it has not yet reached chromosome‐level resolution. A large proportion of predicted proteins remain uncharacterised, restricting the ability to identify effective targets for breeding or chemical control. Compared to other major plant pathogens, Sss remains severely under‐resourced at the molecular level. This paper also summarises studies that have applied molecular tools to investigate resistance in the potato host, revealing early insights but underscoring the need for more extensive research. Overall, this short review identifies key gaps in molecular knowledge and highlights the need for a high‐quality, chromosome‐level reference genome and improved annotation through post‐genomic analyses to support more effective and targeted management strategies for powdery scab disease of potato.

## Graphical abstract

This review consolidates currently available genomic, transcriptomic, proteomic and metabolomic resources for *Spongospora subterranea* f. sp. *subterranea*, revealing substantial gaps that continue to limit molecular understanding of this important potato pathogen. It emphasises the need for a high‐quality, chromosome‐level genome and improved functional annotation, supported by broader downstream analyses, to strengthen resistance research and ultimately enable more effective and targeted management of powdery scab disease in potato.

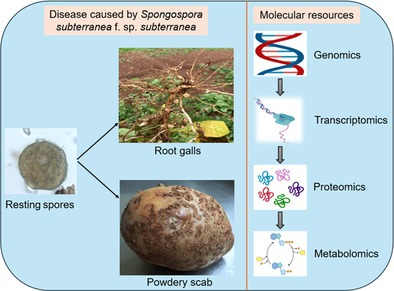

## 1 Introduction

Potato ( *Solanum tuberosum* ) is one of the most widely cultivated food crops globally, providing an important source of nutrition for millions. Potato production faces numerous challenges from diseases that can reduce both yield and quality (Devaux et al. 2021). Among these, powdery scab, caused by infections with the soil‐borne protist *Spongospora subterranea* f. sp. *Subterranea*, is one of the most common and economically important. The disease affects both the quality and marketability of tubers and indirectly reduces yield through root dysfunction (Merz 2008). In addition, Sss is the vector of Potato mop‐top virus (PMTV), compounding its negative impact on crop productivity (Jones and Harrison 1969). Losses due to Sss infection are difficult to quantify and at best estimations. Wilson (2016) reported a putative AUD$16 M p.a. cost of Sss infection to the Australian potato processing sector, and subsequent industry estimates have suggested up to 20% yield loss from the impact of root infection alone (F. Mulcahy, Simplot Australia Pty Ltd.).

As an obligate biotroph, Sss relies on a living host to complete its lifecycle. This pathogen's lifecycle is particularly specialised, involving the production of highly resistant resting spores in the form of sporosori (aggregate of resting spores) that may remain dormant for many years. Dormant spores may be stimulated to germinate by encountering specific chemical signals, such as those found in potato root exudates, which release short‐lived motile zoospores that are chemotaxically attracted to potato roots to which they encyst and infect (Balendres et al. 2016). This ability of Sss to persist in soil over many years has led to infested cropping soils becoming less productive and has largely negated the ability of rotation to impact disease (Harrison et al. 1997).

Although a recent report demonstrated that a combination of soil fumigants can reduce disease incidence and improve yields (Mao et al. 2025), chemical control options for powdery scab remain very limited. Given this, integrated disease management strategies have increasingly focused on breeding resistant potato cultivars (Falloon 2008). Resistance to Sss in potato germplasm is a crucial and sustainable disease management strategy, although no commercial cultivars are completely immune (Merz et al. 2004). Resistance expression can differ between root and tuber infections, requiring assessment at multiple disease stages (Falloon et al. 2016; Merz et al. 2004). Resistance is generally polygenic, complicating breeding efforts aimed at balancing disease resistance with agronomic and market traits (Falloon et al. 2003; Genet et al. 2005; Nitzan et al. 2010). Despite recent efforts focused on Sss resistance breeding, the complexity and polygenic nature of current known resistance has meant selection of highly Sss‐resistant cultivars is yet to be achieved (Falloon et al. 2003; Tegg et al. 2012).

Breeding for resistance is also hampered by limited knowledge of both the pathogen's molecular biology and the host‐pathogen interaction. Identifying resistance genes in the host, as well as critical effector proteins or metabolic targets in the pathogen, requires a solid foundation of molecular data (Nabi et al. 2024). For other major plant pathogens, such foundational resources now include high‐quality reference genomes, gene expression datasets, proteomic profiles, and annotated effector repertoires, tools that have driven rapid progress in understanding pathogen biology and informing control strategies (Prasad et al. 2019; Tintor et al. 2020; Evangelisti and Govers 2024). In contrast, research on Sss still lacks these critical molecular resources. While some progress has been made, most notably the release of draft genomes in 2018 and 2025, these resources remain poorly annotated. Functional genomics, transcriptomics, and proteomics studies are limited in number and scope. This lack of comprehensive molecular data significantly constrains efforts to identify virulence factors, understand resistance mechanisms, or develop molecular diagnostics and fungicide targets (Amezrou et al. 2024; Zhang et al. 2024).

This short review aims to address that gap by bringing together currently available molecular resources for Sss, including genomic, transcriptomic, proteomic, and metabolomic datasets. In doing so, it provides a critical overview of the progress made so far, highlights key limitations of existing data, and identifies areas where further investment is needed. While a recent comprehensive review by Strydom et al. (2024) has provided a broad overview of Sss biology, the present work builds upon and extends this by offering a more focused and technical evaluation of molecular datasets. Specifically, it assesses the quality and completeness of publicly available genomic resources, discusses sequencing methodologies, and identifies critical methodological gaps. Additionally, this study summarises the molecular research focused on the potato host's response to Sss, offering insight into how plant‐based resistance is currently understood. This review aims to define strategic priorities that will accelerate the development of effective, evidence‐based management approaches for potato powdery scab.

## 2 Molecular Resources for *Spongospora subterranea*

The development of molecular resources for Sss has been slow and fragmented, largely due to the biological challenges of working with an obligate biotroph that cannot be cultured independently of its host. However, progress over the past two decades has gradually built a foundation of genomic, transcriptomic, and proteomic datasets that now enable more targeted molecular studies. To assess the current status of these resources, data were compiled from major public repositories, including the National Centre for Biotechnology Information (NCBI), European Nucleotide Archive (ENA), ProteomeXchange, and UniProt. NCBI and ENA were the primary sources for genomic and transcriptomic data, while ProteomeXchange and UniProt provided proteomic datasets. Table 1 summarises all currently available publicly deposited datasets as of June 2025.

**TABLE 1:** Publicly available molecular resources for *Sss*.

Data type	Accession number	Source	Sequencing/Analytical technology	Tissue source	References
Genomic	HO772678‐HO772709	GeneBank	GS FLX 454 Life Sciences System	Potato‐ Sss callus	Burki et al. (2010)
	GU939003‐GU939048	GeneBank	Sanger sequencing	Potato‐ Sss callus	Bulman et al. (2011)
	KF738139	GeneBank	GS FLX 454 Life Sciences System	Sporosori separated from soil	Gutierrez et al. (2016)
	GCA‐900404475 and ERX2560124	GeneBank	Illumina HiSeq 2500 platform	Sporosori from potato tuber	Ciaghi et al. (2018)
	GCA‐049724395 and SRX20863762	GeneBank	Oxford Nanopore MinION; Illumina MiSeq	Potato root galls	Arjarquah et al. (2025)
RNA‐seq	SRX027223	SRA‐NCBI	GS FLX 454 Life Sciences System	Potato‐ Sss callus	Burki et al. (2010)
	ERX943481	SRA‐NCBI	Illumina HiSeq 2500	Potato root galls	Schwelm et al. (2015)
	SRX11490251‐SRX11490256	SRA‐NCBI	Illumina NovaSeq 6000	Potato roots infected with Sss	Balotf et al. (2021a)
	SRX10528964‐SRX10528969	SRA‐NCBI	Illumina NovaSeq 6000	Sporosori from potato tuber	Balotf et al. (2021)
Proteomics	PXD019776	ProteomeXchange	Liquid Chromatography‐Mass Spectrometry (LC–MS/MS)	Sporosori from potato tuber	Balotf et al. (2020)
	PXD027266	ProteomeXchange	LC–MS/MS	Potato roots infected with Sss	Balotf et al. (2021a)
	PXD022089	ProteomeXchange	LC–MS/MS	Sporosori from potato tuber	Balotf et al. (2021b)

The history of molecular research in Sss traces its origins to the early efforts in transcriptome analysis. The first notable step was taken by Burki et al. (2010), who focused on developing an expressed sequence tag (EST) dataset for Sss. Their work resulted in the production of 14,531 contigs, from which they filtered out plant sequences, leaving behind 6249 contigs that were used for phylogenomic screening and gene retrieval. This dataset marked an important step in understanding the genetic makeup of this parasitic protist.

Bulman et al. (2011) introduced an innovative in vitro dual culture system for both Sss and another significant plant pathogen, *Plasmodiophora brassicae* . This system, designed to cultivate both the protists alongside their respective plant hosts, led to the construction of a pilot‐scale DNA library from callus cultures. Surprisingly, almost all clones were derived from Sss rather than the plant host. Through bioinformatic analysis, they uncovered a genome rich in retrotransposable elements, with protein‐coding genes containing introns. Their work also marked a key discovery: the identification of a full‐length non‐LTR retrotransposon, the first transposable element reported in a cercozoan protist, a breakthrough that helped shape future genomic research.

A previous study by Gau et al. (2013) represents one of the first comprehensive population genetic characterisations of Sss, a challenging task due to the lack of suitable genetic markers and the obligate biotrophic nature of the pathogen. Consequently, genotyping efforts have traditionally relied on sequencing housekeeping genes and the internal transcribed spacer (ITS) region. Gau et al. (2013) successfully genotyped 693 individual Sss samples from a wide range of geographic regions, climate zones, potato subspecies, and tissue types, using newly developed polymorphic microsatellite markers. Their analyses demonstrated that South American populations consistently exhibited greater genetic diversity than those from other regions, supporting the hypothesis that South America is the pathogen's centre of origin. Furthermore, estimates of gene flow indicated historic migration from South America to Europe, with Europe subsequently acting as a “bridgehead” for more recent global dissemination. A subsequent study by Muzhinji and van der Waals (2019) focused on 172 samples from South Africa; an additional 27 samples obtained from Colombia were included for comparative purposes. Using six polymorphic microsatellite markers, they detected 75 multilocus genotypes (MLGs), of which only 16 were shared between regions, indicating considerable gene flow and widespread dispersal within South Africa. The presence of identical MLGs in both root‐ and tuber‐derived samples suggested a lack of organ‐specific specialisation. While gene diversity values were relatively low, the population structure showed signs of recombination, either sexual or asexual (Muzhinji and van der Waals 2019). While these findings provided valuable insights, it is now recognised that more robust and detailed population genetic studies require whole‐genome sequencing data, as analyses based solely on ITS or microsatellite markers offer limited resolution of pathogen diversity and evolutionary history (Lu et al. 2025).

In 2016, Sss reached another major milestone when Gutierrez et al. (2016) successfully sequenced the complete mitochondrial genome of this organism. This 37,699 bp mtDNA sequence encodes 16 respiratory chain proteins, 11 ribosomal proteins, and 24 tRNAs. It became the first complete mitochondrial genome from a plasmodiophorid and significantly contributed to refining the evolutionary relationships of this group within the broader Rhizaria supergroup.

The most significant breakthrough came in 2018 with the release of the first draft genome of Sss by Ciaghi et al. (2018). This draft genome, assembled from a single tuber isolate (SSUBK13), spanned 28.08 Mb and was composed of 2340 scaffolds. The assembly included 10,778 predicted genes, with a remarkable 93% completeness according to BUSCO analysis. The draft genome also contained ribosomal DNA and partial mitochondrial sequences, paving the way for further genomic exploration. Despite these advancements, as of Jun 2025, approximately 50% of the protein database for Sss in UniProt remains classified as “uncharacterised protein,” underscoring ongoing challenges in fully elucidating the functional landscape of its genome.

For several years, this draft genome remained the only comprehensive resource available, limiting efforts to understand the organism's biology and pathogenicity at a deeper level. A notable advancement came with the recent publication by Arjarquah et al. (2025), who assembled an improved genome of Sss (isolate SssMN22‐1) from North America. Using Oxford Nanopore long‐read sequencing complemented by Illumina short reads, the genome was assembled to 31.51 Mb with a GC content of 45.7%. The assembly achieved 96.1% completeness based on BUSCO analysis, marking a structural improvement over the 2018 draft. However, despite the increase in assembly quality, the new genome still falls short of a chromosome‐level resolution, consisting of 346 scaffolds. Moreover, it did not significantly enhance the annotation of the Sss genome.

The availability of two genome assemblies, though varied in quality, now provides a more stable foundation for genomic investigations in Sss. The earlier 2018 draft genome (Ciaghi et al. 2018) which was generated using short‐read sequencing technology, limited its contiguity and ability to resolve repetitive regions, characteristics especially problematic for organisms with complex genomic architectures. In contrast, the 2025 genome (Arjarquah et al. 2025) leveraged long‐read sequencing complemented by short reads, resulting in improved scaffold length and overall completeness. This newer assembly enables more comprehensive analyses, such as improved gene prediction, comparative genomics, and preliminary population genomics. However, several key analyses, such as accurate genome synteny, accurate comparative genomics, and the detection of chromosomal rearrangements associated with host adaptation, remain difficult to resolve without a chromosome‐level assembly. The availability of a genome for Sss, despite challenges such as a lack of full annotation, has enabled progress in gene expression analysis. Transcriptome and proteome datasets for Sss are now available, including those detailing transcriptomic changes during spore germination (Balotf, Tegg, et al. 2021) and within both susceptible and resistant host plants (Balotf et al. 2021a). In terms of proteomics, Balotf et al. (2021a, 2021b) reported the first proteome of Sss both in vitro and *in planta*. These studies mark significant advances in understanding Sss. However, despite these efforts, the studies have not identified any specific genes or proteins from Sss that could be used as potential targets for pesticide development.

In summary, while the past 15 years have seen incremental progress in assembling molecular datasets for Sss, the resources remain far behind those available for other major plant pathogens. There remains a need to further improve the genome, ideally toward chromosome‐level resolution, along with additional expression‐based studies to enhance functional annotation and support gene discovery, effector prediction, and the identification of molecular targets for disease control. Bridging this gap is essential for enabling more sophisticated studies into the biology, pathogenicity, and host interaction mechanisms of this persistent and economically significant pathogen.

## 3 Potato Responses to *Spongospora subterranea*

Unlike Sss, potato has benefited from extensive genomic research, culminating in the release of a high‐quality, well‐annotated reference genome (Pham et al. 2020). This resource has paved the way for numerous studies exploring the plant's genetic responses to a range of pathogens, including Sss. Despite this advantage, molecular investigations specifically targeting potato resistance to powdery scab remain limited. Nonetheless, the available studies have provided important preliminary insights into host‐pathogen interactions and potential resistance mechanisms.

Early genomic studies of Sss provided important insights into the pathogen's metabolism and infection biology. Using 454 pyrosequencing, Gutierrez Sanchez et al. (2014) identified genes involved in glycolysis, starch, cellulose, and chitin metabolism, helping to explain how the pathogen survives and develops within potato roots. Building on this, Hernandez Maldonado et al. (2015) showed that resistance in potato can be induced chemically: applications of β‐aminobutyric acid significantly reduced root infection and galling, demonstrating the potential of stimulating host defences to control powdery scab. More recent work has further clarified host‐pathogen interactions. Kamal, Lynch‐Holm, et al. (2024) found that Sss manipulates host starch metabolism during gall development, consuming starch reserves and altering starch‐related gene expression, which may affect host susceptibility. At the same time, innovative biocontrol strategies have been explored: engineered *Bacillus subtilis* strains that secrete the phytocytokine StPep1 substantially reduced root galling and tuber lesions, showing that microbial delivery of immune‐eliciting molecules can enhance host resistance (Moroz et al. 2024).

Recent research has begun to characterise transcriptional responses in potato cultivars with differing susceptibility levels to powdery scab. These studies have identified candidate defence‐related genes and regulatory pathways that may play roles in the early stages of infection. For instance, differential expression of pathogenesis‐related proteins, enzymes involved in cell wall modification, and hormone‐signalling genes has been observed, suggesting that the potato's response to Sss involves a complex network of defence strategies.

In addition to transcriptomic data, proteomic and metabolomic studies have revealed further layers of resistance mechanisms. Defence‐associated proteins, such as those linked to pectin biosynthesis and root‐surface signalling, have been more abundant in resistant cultivars, while metabolite profiling has shown distinct patterns between tolerant and susceptible genotypes. Compounds in root exudates have also been implicated in triggering resting spore germination, highlighting their role in mediating early host‐pathogen interactions. Multi‐omics approaches have further integrated these findings, revealing key pathways such as glutathione metabolism and lignin biosynthesis in resistant cultivars.

Table 2 summarises and integrates key findings from these molecular studies on potato resistance to Sss. Together, they highlight both the progress made and the critical gaps that remain, which must be addressed to inform more effective and targeted breeding strategies for powdery scab resistance.

**TABLE 2:** Molecular studies on potato resistance to powdery scab disease.

Data type	Summary	References
Transcriptomics	Role of salicylic acid in potato resistance to powdery scab caused by Sss. Increased SA levels and enhanced SA signalling reduced pathogen proliferation, highlighting SA's key role in induced resistance.	Jayasinghe et al. (2025)
	Identification of differentially expressed genes in two potato cultivars in response to Sss infection. Defence genes involved in the salicylic acid pathway showed contrasting expression patterns.	Lekota et al. (2019)
Proteomics	Proteomic analysis of 12 potato cultivars. Several proteins were identified more abundant in resistant cultivars. Pectin biosynthesis enzymes were also enriched in resistant cultivars, and pectinase treatment reduced zoospore root attachment.	Yu et al. (2022)
	Proteomics and phosphoproteomics of susceptible and resistant potato cultivars in response to Sss infection. The resistant cultivar showed enhanced defence responses, while the susceptible cultivar exhibited changes related to transporter activity.	Balotf, Wilson, Tegg, et al. (2022)
	The role of root‐surface proteins in potato cultivars resistance to Sss. Differentially abundant proteins were more prominent in resistant cultivars, with proteins like glucan endo‐1,3‐beta‐glucosidase potentially playing a role in regulating zoospore attachment.	Yu et al. (2023)
Metabolomics	Identification of 24 organic compounds in potato root exudates, including amino acids, sugars, and organic acids, that stimulate Sss resting spore germination, leading to earlier zoospore release.	Balendres et al. (2016)
	The role of environmental factors on potato root exudates. Root vigour, cultivar, nutrients, and temperature influenced root exudates, affecting the release of metabolites that stimulate Sss spore germination.	Balendres et al. (2017)
	Untargeted metabolomics of susceptible and tolerant potato cultivars infected by Sss. Tolerant cultivar had higher levels of metabolites related to amino acids, fatty acids, phenolics, and cell wall compounds.	Lekota et al. (2020)
Multi‐omics	Integration of transcriptomic and proteomic identified resistance mechanisms to Sss in potato roots. Glutathione metabolism and lignin metabolic processes were upregulated in the resistant cultivar, while the inositol phosphate pathway was upregulated in the susceptible cultivar. Chromosome 9 of potato genome was identified as a hotspot for glutathione‐S‐ transferase genes.	Balotf, Wilson, Nichols, et al. (2022, 2025)

## 4 Conclusion and Future Directions

The progress of molecular research into Sss and its management remains limited by the need for a more complete and well‐annotated reference genome. This ongoing gap also highlights the importance of expanding transcriptomic and proteomic analyses, which are critical for identifying genes and proteins involved in pathogenicity, host interaction, and potential targets for resistance breeding or pesticide development.

To overcome these challenges, there is a need to produce a chromosome‐scale, fully annotated genome of Sss. Long‐read sequencing technologies such as Oxford Nanopore and PacBio offer promising solutions to address the shortcomings of previous short‐read assemblies. These platforms can generate more contiguous and accurate genome assemblies, better resolving repetitive and complex regions that are typically problematic (Karst et al. 2021). Although the 2025 genome represents a significant improvement, its scaffold‐level resolution (346 scaffolds) limits its utility for investigating higher‐order genome architecture and long‐range synteny. These types of analyses are essential for studying pathogen evolution, host specificity, and adaptation. In other plant‐pathogenic protists, such as *Phytophthora infestans* , chromosome‐scale assemblies have revealed karyotype variation, genome rearrangements, and expression level polymorphisms (Matson et al. 2022). Furthermore, greater assembly contiguity would also support more robust functional annotation by identifying potential regulatory regions and improving syntenic comparisons with related species (Liu et al. 2018). To enable similar insights in Sss, we argue that a chromosome‐scale reference genome, supported by Hi‐C or Pore‐C long‐range scaffolding, remains an essential goal. Moreover, creating a comprehensive pangenome for Sss should be a priority. A pangenome, representing the full genetic repertoire across multiple isolates, would provide critical insights into genetic variation, including core genes conserved across all isolates and accessory genes unique to specific populations or strains (Amir et al. 2020). Understanding this genetic diversity is crucial for elucidating mechanisms of host adaptation, virulence variability, and resistance breakdown, which could inform durable disease management strategies (Salgotra and Chauhan 2023).

Beyond the genome itself, advancing functional annotation is equally critical. Presently, a significant proportion of predicted proteins remain uncharacterised, limiting biological interpretation. Integrating transcriptomic, proteomic, and metabolomic data will be instrumental in assigning functions to these proteins and uncovering key molecular pathways involved in infection and host defence (Depuydt et al. 2023). Such multi‐omics approaches will provide a holistic view of Sss biology and its interactions with the potato host.

The challenges in obtaining high‐quality genomic data persist due to the obligate biotrophic nature of Sss, which complicates DNA extraction from infected plant tissue and environmental samples. These difficulties highlight the need for innovative pathogen isolation and DNA extraction methods. Establishing axenic pathogen co‐cultures with the potato host in tissue culture can remove environmental contaminants; however, pathogen host interactions may not fully reflect natural infection processes (Kamal, Charlton, et al. 2024). While metagenomic approaches have demonstrated that host DNA contamination can be computationally reduced by up to 99% without losing sensitivity (McArdle and Kaforou 2020), in the case of Sss, contamination is not limited to plant material but also includes other microorganisms from soil and plant‐associated microbiomes. Additionally, because the potato genome is substantially larger than that of Sss, host DNA can consume a disproportionate amount of sequencing coverage, limiting the depth of pathogen‐specific data obtained. Future research should focus on developing efficient purification techniques for Sss spores from plant tissue, soil, and other contaminants. Methods such as density gradient centrifugation (Balotf et al. 2020) and filtration (Ciaghi et al. 2018) have shown promise in purifying resting spores from infected tissues. However, due to size variation among Sss sporosori, the efficiency of these methods is not high, and they can only partially purify the spores. Furthermore, combining these purification techniques with optimised DNA extraction protocols, such as enzymatic lysis or chemical treatments tailored for pathogen cells, will be essential for improving DNA quality and facilitating downstream genomic studies.

Overall, the need for a high‐quality Sss genome is not just an academic pursuit; it is fundamental for unlocking the pathogen's genetic secrets and advancing effective control strategies, which will ultimately enable the sustainable management of this pathogen and its devastating effects on potato production. By addressing these knowledge gaps through concerted genomic and molecular efforts, researchers and breeders will be better equipped to understand, predict, and manage Sss infections, safeguarding potato crops for future generations.

## Author Contributions

S.B. and C.W. designed the study. S.B. collected the data and wrote the manuscript. S.B. and C.W. revised and edited this article. All authors read and approved the manuscript.

## Funding

The authors have nothing to report.

## Conflicts of Interest

The authors declare no conflicts of interest.

## Data Availability

Data sharing is not applicable to this article as no datasets were generated during the current study.
